# Effects of Video-Based Patient Education and Consultation on Unplanned Health Care Utilization and Early Recovery After Coronary Artery Bypass Surgery (IMPROV-ED): Randomized Controlled Trial

**DOI:** 10.2196/37728

**Published:** 2022-08-26

**Authors:** Gijs van Steenbergen, Dennis van Veghel, Dideke van Lieshout, Merel Sperwer, Joost ter Woorst, Lukas Dekker

**Affiliations:** 1 Cardiothoracic Surgery Department Catharina Heart Centre Catharina Hospital Eindhoven Netherlands; 2 Dutch Heart Foundation The Hague Netherlands; 3 Department of Biomedical Technology Eindhoven University of Technology Eindhoven Netherlands

**Keywords:** e-Health, eHealth, digital health, patient education, coronary artery bypass surgery, cardiac surgery, health care utilization, costs, cost, economic, coronary, cardiology, heart, surgery, bypass, RCT, randomized controlled trial, video consultation, telehealth, telemedicine, patient-reported, recovery, expense

## Abstract

**Background:**

Health care utilization after coronary artery bypass graft (CABG) surgery is high and is partly of an unplanned nature. eHealth applications have been proposed to reduce care consumption, which involve and assist patients in their recovery. In this way, health care expenses could be reduced and quality of care could be improved.

**Objective:**

The aim of this study was to evaluate if an eHealth program can reduce unplanned health care utilization and improve mental and physical health in the first 6 weeks after CABG surgery.

**Methods:**

A single-blind randomized controlled trial was performed, in which patients scheduled for nonacute CABG surgery were included from a single center in the Netherlands between February 2020 and October 2021. Participants in the intervention group had, alongside standard care, access to an eHealth program consisting of online education videos and video consultations developed in conjunction with the Dutch Heart Foundation. The control group received standard care. The primary outcome was the volume and costs of a composite of unplanned health care utilization, including emergency department visits, outpatient clinic visits, rehospitalization, patient-initiated telephone consultations, and visits to a general practitioner, measured using the Medical Technology Assessment Medical Consumption Questionnaire. Patient-reported anxiety and recovery were also assessed. Intention-to-treat and “users-only” analyses were used.

**Results:**

During the study period, 280 patients were enrolled and randomly allocated at a 1:1 ratio to the intervention or control group. The intention-to-treat analysis consisted of 136 and 135 patients in the intervention and control group, respectively. At 6 weeks, the primary endpoint had occurred in 43 of 136 (31.6%) patients in the intervention group and in 61 of 135 (45.2%) patients in the control group (hazard ratio 0.56, 95% CI 0.34-0.92). Recovery was faster in the intervention group, whereas anxiety was similar between study groups. “Users-only” analysis yielded similar results.

**Conclusions:**

An eHealth strategy comprising educational videos and video consultations can reduce unplanned health care utilization and can aid in faster patient-reported recovery in patients following CABG surgery.

**Trial Registration:**

Netherlands Trial Registry NL8510; https://trialsearch.who.int/Trial2.aspx?TrialID=NL8510

**International Registered Report Identifier (IRRID):**

RR2-10.1007/s12471-020-01508-9

## Introduction

Coronary artery bypass graft (CABG) surgery is one of the most frequently performed cardiac surgeries in the world, which is generally performed with good outcomes and relatively low 30-day mortality (~1.5%) [1]. In more recent years, the care chain for patients undergoing CABG surgery has been demonstrated to increase efficiency and reduce costs. As a result, the duration of hospitalization has decreased substantially, with patients discharged on the 7th postoperative day (mean). These efficiency-driven early discharge protocols require more self-management skills among patients. Early discharge reduces the time physicians can spend with their patients in the direct postoperative phase in spite of the well-known benefit of patient counseling and guidance through recovery [2,3].

After discharge, patients commonly experience anxiety or uncertainty about symptoms or appropriate physical exercise [4]. These issues are typically addressed during hospitalization; however, after discharge, patients’ recall of information is often incomplete and they do not always know who to address with questions [4]. The advantages of a shortened hospital stay might therefore be counterbalanced by preventable unplanned health care utilization, especially since planned care is not initiated until several weeks after discharge. At present, nearly 1 in 7 patients are readmitted in the first 30 days after discharge for noncardiac causes and roughly 15% of patients visit the emergency department within 1 month after CABG surgery [5-8]. It was estimated that potentially preventable readmissions following CABG surgery cost Medicare US $151 million in 2005, placing a significant burden on society [7]. With the expected increase in the number of future patients undergoing CABG surgery, this is a pressing issue urging evaluation and a potential redesign of postoperative follow-up.

eHealth is defined by the World Health Organization as “the cost-effective and secure use of information and communication technologies in support of health and health-related fields,” which encompasses multiple digital interventions that can aid in the delivery of patient-centered care and postoperative patient guidance, thereby potentially reducing unplanned health care utilization [9]. eHealth strategies have been successfully applied in postoperative follow-up in various forms, which have been shown to improve patient outcomes, speed recovery, and reduce health care utilization in various surgical populations [10]. In addition, eHealth has proven to be of value for patients to enhance their self-management through better understanding of their disease, increased independence, and improved acceptance to adhere to lifestyle advice [3,11]. However, experience with eHealth in patients following CABG surgery is limited, and it remains unclear if eHealth strategies would be effective in this population.

The objective of this trial was to fill this knowledge and experience gap. We hypothesized that restructuring the postoperative period with an eHealth strategy will reduce unplanned health care utilization through improved mental and physical health and faster recovery.

## Methods

### Trial Design

The IMPROV-ED trial was a randomized controlled trial (RCT) performed between February 2020 and December 2021 at Catharina Hospital in the Netherlands. A detailed study protocol was published prior to enrollment of the first study participant [11]. No changes were made to the study protocol between publication and initiation of the trial. The trial is reported using the CONSORT (Consolidated Standards of Reporting Trials) checklist for RCTs [12].

### Ethics Considerations

The study was approved by the medical ethics committee (R19.100) and was registered in the Netherlands Trial Registry (NL8510). Written informed consent was obtained from all patients who met the inclusion criteria and were willing to participate.

### Participants

To minimize selection bias, all patients on the waiting list for isolated CABG surgery over 18 years of age were contacted by telephone and informed about the study by one of the investigators. Patients were eligible for participation if they had access to a computer/tablet/smartphone with internet connection and a webcam/built-in camera; had sufficient knowledge of the use of internet and email (assistance was allowed); and were able to speak, read, and interpret the Dutch language. The eHealth strategy would not be applicable to patients who did not comply with these inclusion criteria and they were therefore not eligible for participation. At inclusion, patients were randomized 1:1 to the intervention or control group using a block size of 4. A certified program was used for sequence generation and randomization (Research Manager). When a patient was randomized but no longer qualified for the inclusion criteria or was lost to follow-up, the patient was excluded from further follow-up and analysis.

### Interventions

Patients randomized to the control group received standard postoperative care, comprising planned outpatient follow-up by their cardiothoracic surgeon at 6-8 weeks after discharge and a cardiac rehabilitation program supervised by cardiologists with outpatient follow-up starting between 4 and 8 weeks after surgery. As a result of the COVID-19 health crisis and the measures taken by the government, most of these contacts were telephone consultations (TCs) rather than physical consultations.

Patients randomized to the intervention group had access to the eHealth strategy in addition to standard care. The eHealth strategy comprised web-based educational videos developed by the Dutch Heart Foundation and two postoperative video consultations (VCs) with a physician from the department of cardiothoracic surgery at 1 and 3 weeks after discharge.

Upon randomization, patients in the intervention group received access to the educational videos via a link sent by email. The same link was sent via email again at discharge. By clicking the link, patients were referred to a hidden (for nonparticipants and the control group) part of the website from the Dutch Heart Foundation that contained the educational videos. The content of the educational videos was constructed and validated by physicians and patient representatives prior to the trial. Based on these evaluations and a scoping review of the literature on delivery of information to patients with varying degrees of health literacy, the full content was delivered to patients at inclusion instead of by fragmentized access to videos applicable to the patient’s situation [13]. Nevertheless, to prevent cognitive overload in patients with low health literacy, educational videos were categorized in three categories: treatment (10 videos with information on the surgery and how to prepare for admission), recovery (6 videos about what to expect in the postoperative course and when to contact a physician), and healthy living (2 videos on cardiovascular risk management, including smoking cessation, weight reduction, cholesterol management, and exercise). The videos were delivered in spoken text supported by animations for optimal health communication to patients with low and adequate health literacy [13]. Usage data were extracted from the web log for evaluation purposes. Educational videos were available to patients in the intervention group throughout the trial (ie, not only when the link was sent). See the published study protocol for an illustrative overview of the educational videos [11].

VCs were conducted by a nurse practitioner or junior doctor using Microsoft Teams. The dates for VCs were sent to patients by email at discharge. On the day of the VC, patients received an email with a link providing access to the VC. The VC was not scheduled on the same day as routine outpatient follow-up. During the VCs, patients were questioned about their recovery and physical and mental complaints. The sternotomy wound was visually inspected. Patients who required physical examination or diagnostic tests based on the VC were instructed to visit the general practitioner or emergency department, or were scheduled for early outpatient follow-up (within 1 week) at discretion of the physician. The nurse practitioner/junior doctor who conducted the VCs was blinded to the study’s objectives and outcomes. Study participants were not blinded. If a VC was unexpectedly not possible (eg, due to unforeseen connection errors, problems with hardware, technical issues), the VC was replaced by a TC. Reasons for replaced VC were reported.

### Outcomes

The primary outcomes of the IMPROV-ED trial were the volume and costs of unplanned health care utilization defined by a composite of all emergency department visits, outpatient clinic visits, rehospitalization, patient-initiated TCs, and visits to a general practitioner, as measured by the Institute for Medical Technology Assessment Medical Consumption Questionnaire (iMCQ) at the 6-week follow-up [14]. Cross-validation with the patients’ reported health care utilization was performed by contacting their health care providers. The secondary outcomes were the individual unplanned health care activities, and a composite of planned and unplanned in-hospital care (emergency department visits, outpatient clinic visits, rehospitalization, and patient-initiated TCs) and planned and unplanned primary care (consultations with a general practitioner, allied health professionals, psychologists) at 6 weeks. The other secondary outcomes were the patients’ self-reported physical and mental health, as measured with the Hospital Anxiety and Depression Scale (HADS) and Recovery Index-10 (RI-10) questionnaires [15,16].

### Data Collection

All patients received questionnaires at inclusion (anxiety subscale of the HADS), at discharge (HADS and RI-10), 1 week after discharge (HADS and RI-10), 2 weeks after discharge (HADS and RI-10), and 6 weeks after discharge (HADS, RI-10, and iMCQ). Only the anxiety subscale from the HADS was used. A higher score indicated more symptoms of anxiety (HADS maximum score 21) or favorable progress of recovery (RI-10 maximum score 50). The iMCQ resulted in absolute frequencies of visits for the questioned care activities. Patients in the intervention group also received a self-made questionnaire to evaluate the eHealth strategy and to question them about the use of the education videos (see Figures S1 and S2 in Multimedia Appendix 1). If patients had not returned the iMCQ by 8 weeks postdischarge, the questionnaire was conducted over the telephone. If patients had not returned 2 subsequent questionnaires, a research nurse called patients with a reminder. Questionnaires that were not returned or collected otherwise were considered missing.

### Statistical Analysis

We calculated the sample size needed for the study based on the expected effect of the intervention on the primary outcome. Previous studies using a comparable eHealth strategy in CABG patients with health care utilization measured with the iMCQ were not available. In a study with abdominal surgery patients, total health care utilization was estimated at a mean of 0.88 (SD 0.15) per patient [17]. In a systematic review by van der Meij et al [10], the effect of an eHealth strategy in surgical patients was not consistent. Therefore, a small to medium effect (*d*=0.35) was expected from our intervention. Combined with an α of .05 and a power of 0.80, a total sample size of 260 patients was required. We aimed for 280 participants to account for loss to follow-up and nonadherence to the intervention and return of questionnaires (attrition rate 5%, rounded up to a whole number). Demographic data of randomized patients were collected using definitions in line with the Netherlands Heart Registration [18]. Education was grouped into three levels (low, medium, and high) according to the general definition by Statistics Netherlands (see Multimedia Appendix 1 for the full definition).

The main analysis was performed according to the intention-to-treat (ITT) principle. Because patients in the intervention group were not obliged to use the educational videos and VCs might not be possible due to technical errors, per-protocol analysis was also performed, which included only patients who used the intervention strategy as intended (defined as having at least one VC or TC and accessed the educational videos at least once).

Planned subgroup analyses of the primary outcome were performed according to age (<65 years vs ≥65 years), sex, recent myocardial infarction, left ventricular function, diabetes, type of CABG (on-pump vs off-pump), log EuroScore, and highest level of education.

Continuous variables and outcomes are expressed as mean (SD) in cases of a normal distribution and as median (IQR) in cases of a nonnormal distribution. The Kolmogorov-Smirnov test and Q-Q plots were used to test for normality of the data distribution. Categorical data are summarized as absolute and relative frequencies. The updated Dutch Manual for Cost Analysis in Health Care Research was used as the source for cost prices per health care activity if available [19]. Other tariffs were calculated using top-down microcosting as described by Tan and Hakkaart-van Roijen et al [20, 21] (see Multimedia Appendix 1 for details). Each consumed health care activity was multiplied by the cost price and total costs were calculated by summing these multiplications. The HADS and RI-10 questionnaire scores at each interval were compared between study groups. *P*<.05 was considered significant. Primary and secondary outcomes are presented with effect-size estimates and 95% CIs using the Cox proportional hazards model. The proportional hazard assumption was assessed by log (–log) plots. Analyses were performed using SPSS 25 and RStudio.

## Results

### Study Population

**Figure 1:**
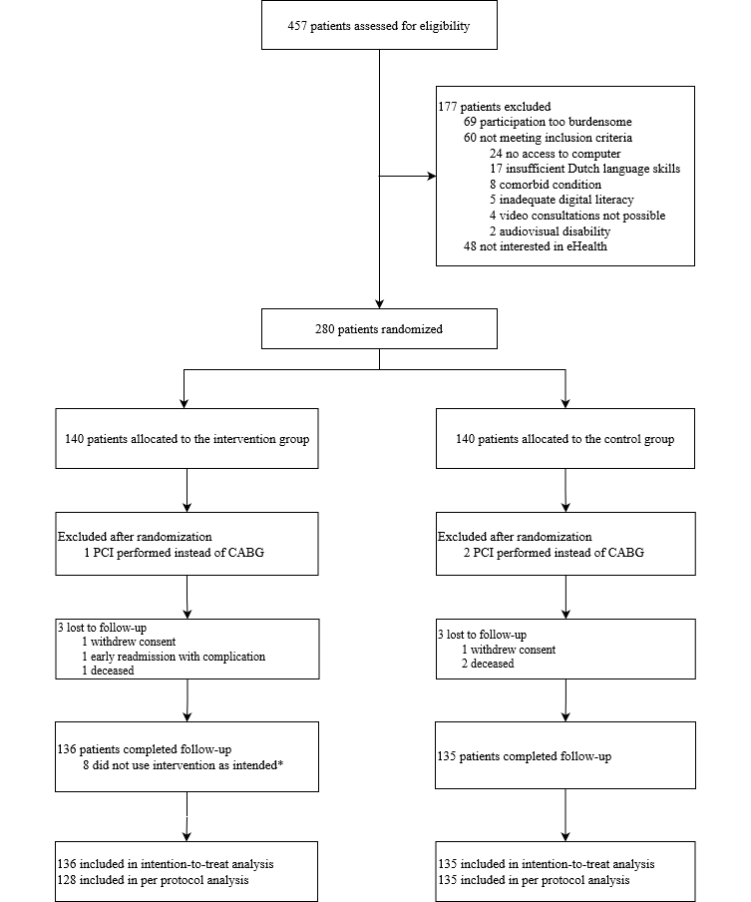
Enrollment overview. CABG: coronary artery bypass graft; PCI: percutaneous coronary intervention.

In total, 280 patients were included in the study between February 2020 and December 2021, and subsequently randomized yielding 140 patients in each study group. One patient in the intervention group and two patients in the control group were excluded after randomization because they underwent percutaneous coronary intervention instead of CABG surgery. In the intervention group, three patients were lost to follow-up (1 withdrew consent, 1 had an early readmission due to a complication, and 1 died). In the control group, three patients were lost to follow-up (1 withdrew consent and 2 died). The ITT analysis therefore consisted of 136 and 135 patients in the intervention and control group, respectively. Weblog and planning data revealed that 8 patients did not use the intervention as intended, whereby 128 patients were included in the intervention group in the per-protocol analysis (Figure 1).

Baseline characteristics of patients were similar in the two groups (Table 1), with a median age of 67.9 and 69.6 years for the intervention and control group, respectively. The majority of patients were male in both groups. At the time of surgery, 25% of patients had an urgent indication and the remainder underwent surgery in the elective setting. In the majority of patients, on-pump CABG was performed using 3 distal anastomoses. The left or right internal mammary artery was used in >98% of patients. Duration of admission was also similar in the two groups (Table 1).

**Table 1:** Baseline characteristics and procedural data (intention-to-treat analysis).

Characteristics		eHealth group (n=136)	Standard care (n=135)
Age (years), median (IQR)		67.9 (61.5-73.3)	69.6 (65.2-74.1)
Male, n (%)		121 (89.6)	113 (83.1)
BMI, median (IQR)		27.7 (25.1-30.6)	27.2 (25.2-30.3)
**Medical history, n (%)**			
	Diabetes mellitus	45 (33.3)	33 (24.3)
	Chronic pulmonary disease	7 (5.2)	15 (11.0)
	Atrial fibrillation	9 (6.7)	6 (4.4)
	Multivessel disease	117 (86.7)	121 (89.0)
	Peripheral vascular disease	17 (12.5)	17 (12.6)
	Renal impairment (MDRD^a^<60 mL/min/1.73 m^2^)	10 (7.4)	1 (8.1)
	Previous stroke	3 (2.2)	5 (3.7)
	Recent MI^b^ (90 days)	45 (33.3)	46 (33.8)
	Previous PCI^c^	36 (26.5)	31 (22.9)
Left ventricular ejection fraction, median (IQR)		55 (50-55)	55 (50-55)
Ejection fraction≤30%, n (%)		0 (0)	3 (2.2)
NYHA^d^ class>II, n (%)		3 (2.2)	7 (5.2)
**Current health status**			
	SF-36^e^ physical score, median (IQR)	51 (43-56)	48 (40-51)
	SF-36 mental score, median (IQR)	58 (55-63)	59 (55-64)
	HADS^f^, median (IQR)	3 (1-7)	3 (1-6)
**Level of education, n (%)**			
	Low	36 (26.5)	42 (31.1)
	Intermediate	53 (39.0)	55 (40.7)
	High	47 (34.6)	38 (28.1)
**Procedural data**			
	EuroSCORE log, median (IQR)	2.40 (1.82-4.06)	2.87 (2.01-4.28)
	EuroSCORE II, median (IQR)	1.41 (1.05-2.04)	1.32 (0.78-2.43)
	Use of ECC^g^, n (%)	110 (81.5)	101 (74.8)
	ECC duration in users (min), median (IQR)	74 (60-91)	76 (64-91)
	Number of distal anastomoses, median (IQR)	3 (2-4)	3 (2-4)
	Hospital stay (days), median (IQR)	6 (5-7)	6 (5-7)

^a^MDRD: Modification of Diet in Renal Disease.

^b^MI: myocardial infarction.

^c^PCI: percutaneous coronary intervention.

^d^NYHA: New York Heart Association.

^e^SF-36: Short Item-36.

^f^HADS: Hospital Anxiety and Depression Scale.

^g^ECC: extracorporeal circulation.

### Outcomes

At 6 weeks, care was consumed by less patients in the intervention group than in the control group (Table 2). The benefit of the eHealth strategy was most noticeable in patients over 65 years of age, those of male sex, those with recent myocardial infarction, or with a EuroScore>2 (see Figure S3 in Multimedia Appendix 1). Reduction in individual care activities was significantly different between groups for TCs and was borderline significant for general practitioner visits (Table 2). Costs related to the primary outcome were significantly higher in the standard care group compared with those in the eHealth group (*P*<.001, Table 2), which was attributed to the higher volume of care consumption in the control group (see Table S1 in Multimedia Appendix 1).

A composite of unplanned in-hospital care, a composite of planned and unplanned in-hospital care after discharge, and use of planned and unplanned primary care were all higher in the control group than the intervention group (Table 2). The volume of consumed care was also higher in the control group (Table S1 of Multimedia Appendix 1).

**Figure 2:**
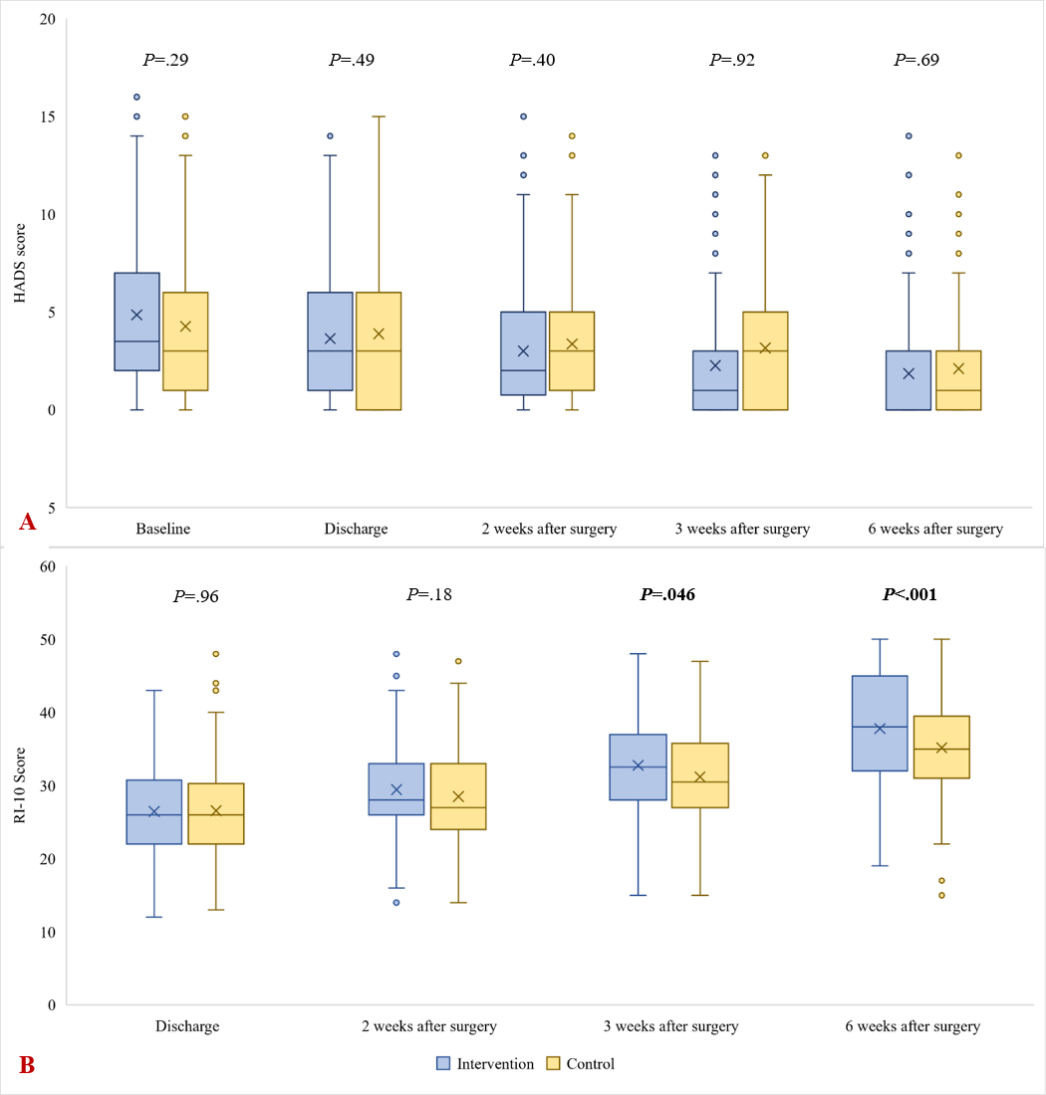
Anxiety level measured with the Hospital Anxiety and Depression Scale (HADS) anxiety subscale (A) and progress of recovery measured using the recovery index-10 (RI-10) questionnaire (B).

The RI-10 score, indicating patient-reported recovery, was significantly higher in the intervention group in the 3rd and 6th weeks after discharge (Figure 2). Anxiety was not significantly different between study groups (Figure 2). Per-protocol analysis revealed similar findings (see Tables S2 and S3 in Multimedia Appendix 1).

**Table 2:** Outcomes at 6 weeks.

Outcomes		eHealth group (n=136)	Standard care (n=135)	Hazard ratio (95% CI)	*P* value
**Primary outcomes**					
	Composite outcome^a^, n (%)	43 (31.6)	61 (45.2)	0.56 (0.34-0.92)	.02
	Cost (Euro^b^), Median (IQR)	0 (0-95)	66 (0-215)	N/A^c^	<.001
	Cost (Euro), mean (SD)	183 (515)	285 (777)	N/A	<.001
**Secondary outcomes, n (%)**					
	Composite unplanned in-hospital care	36 (26.5)	53 (39.3)	0.56 (0.33-0.93)	.03
	Emergency department visits	14 (10.3)	23 (17.0)	0.56 (0.27-1.14)	.11
	Readmissions	7 (5.1)	9 (6.7)	0.76 (0.28-2.10)	.59
	Outpatient clinic visits	11 (8.1)	10 (7.4)	1.10 (0.45-2.68)	.83
	Telephone consultations	29 (21.3)	47 (34.8)	0.51 (0.29-0.87)	.01
	General practitioner visits (unplanned)	28 (20.6)	41 (30.4)	0.59 (0.34-1.04)	.07
	Composite of all in-hospital care^d^	69 (50.7)	97 (71.9)	0.40 (0.24-0.67)	<.001
	Composite of all primary care^e^	82 (60.3)	101 (74.8)	0.58 (0.36-0.97)	.04

^a^Composite of unplanned health care utilization (ie, emergency department visits, readmissions, outpatient clinic visits, telephone consultations, or general practitioner visits).

^b^1 Euro=US $1.13.

^c^N/A: not applicable.

^d^Composite of in-hospital care comprising planned and unplanned emergency department visits, readmissions, outpatient clinic visits, and telephone consultations.

^e^Composite of primary care comprising planned and unplanned visits to the general practitioner, visits to allied health professionals (physical therapists, dieticians, speech therapists, exercise therapy, social workers), and psychologist visits.

### Process Evaluation

Among patients who were provided access to the educational videos, 95% accessed the videos at least once. A total of 248 of the 272 planned VCs were conducted. Eight patients did not use the intervention as intended and did not receive VCs (n=16 VCs). The other VCs that did not take place were substituted with a TC due to technical errors (n=8). The median duration of VCs was 10 minutes (IQR 8-11) for the first VC and was 8 minutes (IQR 7-9) for the second VC. Patients reported positive attitudes toward the education videos and the VC (Figures S1 and S2 in Multimedia Appendix 1). Most notably, patients reported a fairly positive attitude toward substitution of a face-to-face contact with a VC, but patients also reported that the VC with a physician (not the surgeon) or nurse practitioner should not substitute the consultation with the surgeon.

## Discussion

### Principal Findings

The principal finding of the IMPROV-ED trial is that an eHealth strategy comprising educational videos and VCs results in a reduction of unplanned care and costs. In addition, the eHealth strategy is associated with faster patient-reported recovery. These findings are of medical and societal importance given the increasing interest in digital health and the need for value-based alongside evidence-based care. Our study is the first to provide robust evidence that an eHealth intervention can aid in reduction of health care utilization and associated costs. This effect appears applicable to both in-hospital care as well as primary care. One of the most pressing concerns from health care insurance companies and decision-makers toward eHealth is the great investment that is required for development of content and the necessary infrastructure and issues that arise after implementation due to lack of reimbursement options [22]. Our findings refute these concerns by showing positive effects on costs. Furthermore, the eHealth strategy did not only contribute to less patients consuming care (Table 2) but also reduced the care consumed per patient (Table S1 of Multimedia Appendix 1), which underlines the high potential of eHealth strategies for this patient population to also positively influence the burden on health care personnel. With an aging population, a vast increase in health care consumption is expected in the near future. Based on the results of our study, an eHealth program is proven to aid in the sustainment of health care systems.

The findings of our study shine new light on previous studies comparing an eHealth program with standard care because it is the first study to use health care utilization as a primary outcome [10]. Very limited studies are available that use health care utilization as an outcome, and those that have considered care consumption as a secondary or tertiary outcome. Previous studies were also not adequately powered to draw reliable conclusions on the use of eHealth in reduction of care consumption and, consequently, these studies reported mixed outcomes. For example, Keeping-Burke et al [23] incorporated health care use as a tertiary outcome in an RCT of patients after CABG surgery using postoperative VCs, and concluded that patients in the telehealth group had fewer physician contacts. Zahlmann et al [24] used telecommunication in the postoperative period after cataract surgery (n=62) and also concluded that care in the intervention group was lower than that in the control group. Conversely, Barnason et al [25,26] conducted two RCTs in 232 and 50 CABG patients, respectively, using a supportive telehealth program and concluded that both groups had similar health care use at 6-month follow-up. Barnason et al [25] and Keeping-Burke et al [23] both reported no differences in emergency department visits and readmissions between study groups. Readmission was also similar in a study by Gandsas et al [27] after laparoscopic gastric bypass using robotic telerounding during admission.

Another major strength of the current eHealth program is that it provides patients of various degrees of socioeconomic status and health literacy with information on the procedure and their medical condition from a reliable source that is endorsed by their surgeon. The Dutch Heart Foundation is a respected organization that is dedicated to providing information on cardiovascular health, advocating patient interests, and conducting research [28]. The educational videos are developed in conjunction with patient representatives and physicians. In the VCs, additional questions are answered and uncertainties are addressed. The impact of educational videos and VCs is presumably in improvement of self-management skills and reduction of fear and anxiety. Recall of information on information provided preoperatively or at discharge is often incomplete, and patients might not know what physical activity is allowed after discharge or who to contact in case of complaints. Patients can turn to the internet for information; however, this information is uncontrolled, sometimes inaccurate, and is not tailored to the care processes of their provider. Because planned care is not initiated until 6 weeks after surgery (and sometimes later in practice), conflicting advice can induce insecurity, which will lead to use of care and will hamper recovery. The results of our study are consistent with this hypothesis. Nevertheless, the anxiety symptoms measured with the HADS questionnaire relate to anxiety in a narrow sense, whereas the anxiety experienced by patients after CABG surgery is likely to be more subtle in nature, which may have contributed to the nonsignificant difference in measured anxiety found in this study.

However*,* health care utilization is the resultant of a multifactorial behavioral model that attributes a combination of predisposing factors (eg, patient characteristics such as age, sex, sociodemographic parameters, or health literacy and attitude toward health), enabling factors (eg, income, health insurance status, health care organization), and need factors (eg, experience with health care) to health care utilization [29]. The eHealth strategy used in the IMPROV-ED trial has a positive influence on some of these attributes but not all. Interestingly, subgroup analysis showed that the eHealth program had a greater benefit in more vulnerable patients (EuroScore≥2) and revealed a trend toward more benefit in patients with a low level of education. By contrast, a small group of patients who provided informed consent did not use the educational videos or VCs that were part of the eHealth strategy. These patients reported to have received sufficient information from their physician, nurse, or paramedic during admission, or that they found the relevant information online themselves. It might therefore be reasonable to consider adding different modes of digital health delivery to the currently used eHealth strategy (eg, mobile apps, live chat, home monitoring, telerehabilitation) to manage more attributes of health utilization and to offer a more individualized approach tailored to the patients’ needs. Combining different modes of digital care might thereby further reduce health care utilization and potentially also improve clinical outcomes [22].

### Learning Points and Limitations

Even though the IMPROV-ED study yielded positive results toward the primary outcome (Table 2) and patients were generally positive about the eHealth strategy (Figures S2 and S3 in Multimedia Appendix 1), several learning points and limitations should be taken into account for future eHealth programs.

First, the IMPROVE-ED trial is designed for patients who consume care as a result of insecurity, anxiety, lack of medical knowledge, and/or inadequate discharge counseling. As can be concluded from Figure 1, a relevant number of patients who were invited to participate in the trial did not provide informed consent due to the general burden of having to undergo cardiac surgery (patients used terms such as “stressful,” “anxiety,” and “insecurity”) in conjunction with study obligations. The effect of the eHealth strategy may be underestimated because this group of patients might have been part of the target population in which the eHealth strategy would have incremental value. Due to ethical constraints (patients did not provide informed consent for participation and thus for data collection), these patients were not further analyzed for the study outcomes.

In this study, standard care was not replaced by digital alternatives, and yet the costs of the intervention group were still lower than those of the control group receiving only standard care. Because VCs were used as an add-on to standard care, there are potentially more opportunities to reduce costs further. The fact that eHealth is being implemented *on top* of current health care services is, in addition to cost concerns, one of the challenges identified by the European Society of Cardiology as hampering the introduction of eHealth into everyday clinical practice [22]. Future endeavors should focus on investigating the potential of substitution of standard physical care with digital alternatives, especially since the patients’ attitude was generally positive toward the (hypothetical) substitution of a physical contact with a VC in this study (Figure S1 of Multimedia Appendix 1). Previous studies also stated that it is feasible to obtain the same effective communication and interaction with VCs as with face-to-face care [30].

The majority of patients included in the IMPROV-ED trial were included during the COVID-19 pandemic. The results of the study might therefore be an underrepresentation of care consumption because patients feared transmission in the hospital setting [31]. Nevertheless, the randomized design balances this influence between the study groups.

### Conclusion

An eHealth strategy comprising educational videos and VCs can reduce unplanned in-hospital and primary health care utilization and costs, and can aid in faster patient-reported recovery following CABG surgery.

## Appendix

Multimedia Appendix 1 Definitions, tariffs for consumed care, and supplementary data (Tables S1-S3, Figures S1-S3).

Multimedia Appendix 2 CONSORT-eHEALTH checklist (V 1.6.2).

## Abbreviations

CABG: coronary artery bypass graft
CONSORT: Consolidated Standards of Reporting Trials
HADS: Hospital Anxiety and Depression Scale
iMCQ: Institute for Medical Technology Assessment Medical Consumption Questionnaire
ITT: intention to treat
RCT: randomized controlled trial
RI-10: Recovery Index-10
TC: telephone consultation
VC: video consultation
