# Supplementary material for: Effects of Video-Based Patient Education and Consultation on Unplanned Health Care Utilization and Early Recovery After Coronary Artery Bypass Surgery (IMPROV-ED): Randomized Controlled Trial
Source: J Med Internet Res. 2022 Aug 26;24(8):e37728. doi: 10.2196/37728 (PMC9463622; doi:10.2196/37728)
Supplement: Multimedia Appendix 1 [file jmir_v24i8e37728_app1.docx]

**SUPPLEMENTAL MATERIAL**

CONTENT

[**DEFINITIONS** 2](#_Toc94094561)

[Education level 2](#_Toc94094562)

[**TARIFFS FOR CONSUMED CARE** 3](#_Toc94094563)

[**TABLES** 4](#_Toc94094564)

[Table 1. Volume and costs of outcomes at 6 weeks 4](#_Toc94094565)

[Table 2. Baseline characteristics and procedural data (per protocol analysis) 5](#_Toc94094566)

[Table 3. Outcomes at 6 weeks (per-protocol analysis) 6](#_Toc94094567)

[**FIGURES** 7](#_Toc94094568)

[Figure 1. Subgroup analysis 7](#_Toc94094569)

[Figure 2. Patient feedback on education videos 8](#_Toc94094570)

[Figure 3. Patient feedback on video consultations 9](#_Toc94094571)

# DEFINITIONS

## Education level

‘’For highest level of education, the International Standard Classification of Education was used as the basis. Three levels are distinguished: lower, medium and high education level. The lower education level includes Groups 1 through 8 (all years) of primary and special primary education plus the first three years of senior general secondary education (HAVO) and pre-university secondary education (VWO); the various pathways of prevocational secondary education (VMBO) including lower secondary vocational training and assistant’s training (MBO-1). The medium education level includes upper secondary education (HAVO/VWO), basic vocational training (MBO-2), vocational training (MBO-3), and middle management and specialist education (MBO-4). Higher education refers to associate degree programmes, higher education (HBO/WO) Bachelor programmes; 4-year education at universities of applied sciences (HBO); Master degree programmes at universities of applied sciences and at research universities (HBO, WO); and doctoral degree programmes at research universities (WO). ‘’

Source : <https://www.cbs.nl/en-gb/news/2018/20/well-being-not-distributed-equally/education-level>

Accessed 01 February 2020

# TARIFFS FOR CONSUMED CARE

| **Care activity** | **Tariff in euro (€)*** | **Source** |
| --- | --- | --- |
| Emergency department visit | € 259 | Dutch Manual for Cost Analysis in Health Care Research^19^ |
| Readmission | € 476 |  |
| Outpatient clinic visit | € 91 |  |
| General practitioner visit | € 33 |  |
| Allied health professionals |  |  |
| Physical therapy | € 33 |  |
| Exercise therapy | € 34 |  |
| Speech therapy | € 30 |  |
| Dieticians | € 32 |  |
| Social worker | € 65 |  |
| Psychologist visit | € 64 |  |
| Telephonic consultation | € 42 | Top down micro costing^§^ |
| Video consultations | € 21 | Top down micro costing^§^ |

*Prices are per visit/day

§ Tow down micro costing for each care activity was based on personnel costs, housing-, depreciation- and overhead surcharge according to the method of the costs manual of the Netherlands Healthcare Institute.^20,21^ The difference in costs between a telephone consultation and a video consultation is mainly driven by the healthcare professional who performs the consultation (medical specialist vs. nurse practitioner/junior doctor).

# TABLES

## **Table 1.** Volume and costs of outcomes at 6 weeks

|  | **eHealth group**  N = 136 | | |  | **Standard care**  N = 135 | | |  |  |
| --- | --- | --- | --- | --- | --- | --- | --- | --- | --- |
|  | No. visits among those visiting |  | Costs in € |  | No. visits among those visiting |  | Costs in € |  | P-value for € |

|  | Median (IQR) |  | Median (IQR) | Mean ± SD |  | Median (IQR) |  | Median (IQR) | Mean ± SD |  |  |
| --- | --- | --- | --- | --- | --- | --- | --- | --- | --- | --- | --- |
| **Primary outcomes** |  |  |  |  |  |  |  |  |  |  |  |
| Composite outcome † | 2 (1– 4) |  | 0 (0 – 99) | 183 ± 515 |  | 2 (1 – 4) |  | 42 (0 – 150) | 285 ± 777 |  | < 0.01 |
|  |  |  |  |  |  |  |  |  |  |  |  |
| **Secondary outcomes** |  |  |  |  |  |  |  |  |  |  |  |
| Composite unplanned in-hospital care | 1 (1 – 2) |  | 0 (0 – 91) | 185 ± 521 |  | 2 (1 – 3) |  | 0 (0 – 182) | 298 ± 777 |  | < 0.01 |
| Emergency department visits | 1 (1 – 1) |  | 0 (0 – 0) | 32 ± 107 |  | 1 (1 – 2) |  | 0 (0 – 0) | 61 ± 162 |  | 0.129 |
| Readmissions | 1 (1 – 1) |  | 0 (0 – 0) | 112 ± 495 |  | 1 (1 – 1) |  | 0 (0 – 0) | 165 ± 698 |  | 0.586 |
| Outpatient clinic visits | 1 (1 – 1) |  | 0 (0 – 182) | 7 ± 25 |  | 1 (1 – 2) |  | 0 (0 – 182) | 11 ± 43 |  | 0.088 |
| Telephonic consultations | 1 (1 – 2) |  | 0 (0 – 0) | 15 ± 34 |  | 2 (1 – 2) |  | 0 (0 – 42) | 28 ± 44 |  | < 0.01 |
| General practitioner visits | 1 (1 – 2) |  | 0 (0 – 0) | 16 ± 37 |  | 1 (1 – 2) |  | 0 (0 – 33) | 20 ± 33 |  | 0.037 |
|  |  |  |  |  |  |  |  |  |  |  |  |
| Composite of all in-hospital care § | 2 (1 – 3) |  | 42 (0 – 259) | 255 ± 545 |  | 2 (1 – 3) |  | 168 (0 – 273) | 370 ± 796 |  | 0.014 |
| Including VCs |  |  | 84 (42 – 301) | 297 ± 545 |  |  |  | 168 (0 – 273) | 370 ± 796 |  | < 0.01 |
| Composite of all primary care‡ | 2 (1 – 4) |  | 0 (0 – 33) | 20 ± 42 |  | 2 (1 – 4) |  | 0 (0 – 66) | 47 ± 84 |  | < 0.01 |

† The primary outcome was a composite of unplanned healthcare utilisation, i.e. emergency department visits, readmissions, outpatient clinic visits, telephonic consultations or general practitioner visits

§ The composite of in-hospital care consisted of planned and unplanned emergency department visits, readmissions, outpatient clinic visits and telephonic consultations

‡ The composite of primary care consisted of planned and unplanned visits to the general practitioner, visits to allied health professionals (physical therapists, dieticians, speech therapists, ergometrists, social workers) and psychologist visits.

## **Table 2.** Baseline characteristics and procedural data (per protocol analysis)

|  | **eHealth group**  N = 128 | **Standard care**  N = 135 | P-value |
| --- | --- | --- | --- |
| **Patient characteristic** |  |  |  |
| Age (years) – median (IQR) | 67.2 (61.2 – 72.9) | 69.6 (65.2 – 74.1) | 0.13 |
| Male – no. (%) | 114 (89.1) | 113 (83.1) | 0.16 |
| Body-mass index – median (IQR) | 27.4 (24.9 – 30.4) | 27.2 (25.2 – 30.3) | 0.71 |
| Medical history – no./total no. (%) |  |  |  |
| Diabetes Mellitus | 41 (32.0) | 33 (24.3) | 0.22 |
| Chronic pulmonary disease | 7 (5.5) | 15 (11.0) | 0.18 |
| Atrial fibrillation | 9 (7.0) | 6 (4.4) | 0.43 |
| Multi vessel disease | 111 (86.7) | 121 (89.0) | 0.71 |
| Peripheral vascular disease | 16 (12.5) | 17 (12.6) | 1.00 |
| Renal impairment (MDRD<60 mL/min/1.73 m2) | 10 (7.8) | 1 (8.1) | 1.00 |
| Previous stroke | 3 (2.3) | 5 (3.7) | 0.72 |
| Recent MI (90 days) | 42 (32.8) | 46 (33.8) | 0.89 |
| Previous PCI | 32 (25.0) | 31 (22.9) | 0.77 |
| Left ventricular ejection fraction – median (IQR) | 55 (50 – 55) | 55 (50 – 55) | 0.21 |
| Ejection fraction ≤ 30% - no. (%) | - | 3 (2.2) | 0.25 |
| NYHA class > II – no. (%) | 2 (1.6) | 7 (5.2) | 0.16 |
| Current health status |  |  |  |
| SF-36 physical score – median (IQR) | 49 (41 – 54) | 48 (40 – 51) | 0.67 |
| SF-36 mental score– median (IQR) | 56 (52 – 61) | 59 (55 – 64) | 0.26 |
| HADS – median (IQR) | 3 (1 – 6) | 3 (1 – 6) | 0.84 |
| Level of education |  |  | 0.34 |
| Low | 34 (26.6) | 42 (31.1) |  |
| Intermediate | 54 (42.2) | 55 (40.7) |  |
| High | 40 (31.3) | 38 (28.1) |  |
| **Procedural data** |  |  |  |
| EuroSCORE log – median (IQR) | 2.29 (1.81 – 3.61) | 2.87 (2.01 – 4.28) | 0.18 |
| EuroSCORE II – median (IQR) | 1.15 (0.76 – 2.23) | 1.43 (1.10 – 2.39) | 0.22 |
| Use of ECC – no. (%) | 107 (83.6) | 101 (74.8) | 0.11 |
| ECC duration in users (min) – median (IQR) | 75 (60 – 91) | 76 (64 – 91) | 0.83 |
| No. of distal anastomoses – median (IQR) | 3 (2 – 4) | 3 (2 – 4) | 0.34 |
| Hospital stay (days) – median (IQR) | 6 (5 – 7) | 6 (5 – 7) | 0.88 |

## **Table 3**. Outcomes at 6 weeks (per-protocol analysis)

|  | **eHealth group**  N = 136 | **Standard care**  N = 135 | Hazard ratio (95% CI) | P-value |
| --- | --- | --- | --- | --- |
| **Primary outcomes, no. (%)** |  |  |  |  |
| Composite outcome † | 42 (32.8) | 61 (45.2) | 0.59 (0.36 – 0.98) | 0.044 |
|  |  |  |  |  |
| **Costs, €** |  |  |  |  |
| Primary outcome |  |  |  |  |
| Median (IQR) | 0 (0 – 119) | 66 (0 – 215) |  | < 0.01 |
| Mean ± SD | 169 ± 456 | 285 ± 777 |  |  |
|  |  |  |  |  |
| **Secondary outcomes, no. (%)** |  |  |  |  |
| Composite unplanned in-hospital care | 35 (27.3) | 53 (39.3) | 0.58 (0.35 – 0.98) |  |
| Emergency department visits | 13 (10.2) | 23 (17.0) | 0.57 (0.28 – 1.19) |  |
| Readmissions | 6 (4.7) | 9 (6.7) | 1.10 (0.45 – 2.68) |  |
| Outpatient clinic visits | 11 (8.6) | 10 (7.4) | 0.69 (0.43 – 1.15) |  |
| Telephonic consultations | 28 (21.9) | 47 (34.8) | 0.54 (0.30 – 0.91) |  |
| General practitioner visits | 27 (21.1) | 41 (30.4) | 0.61 (0.35 – 1.07) |  |
|  |  |  |  |  |
| Composite of all in-hospital care § | 66 (51.6) | 97 (71.9) | 0.42 (0.25 – 0.69) |  |
| Composite of all primary care‡ | 75 (58.6) | 101 (74.8) | 0.62 (0.37 – 1.03) |  |

† The primary outcome was a composite of unplanned healthcare utilisation, i.e. emergency department visits, readmissions, outpatient clinic visits, telephonic consultations or general practitioner visits

§ The composite of in-hospital care consisted of planned and unplanned emergency department visits, readmissions, outpatient clinic visits and telephonic consultations

‡ The composite of primary care consisted of planned and unplanned visits to the general practitioner, visits to allied health professionals (physical therapists, dieticians, speech therapists, ergometrists, social workers) and psychologist visits.

# FIGURES

**
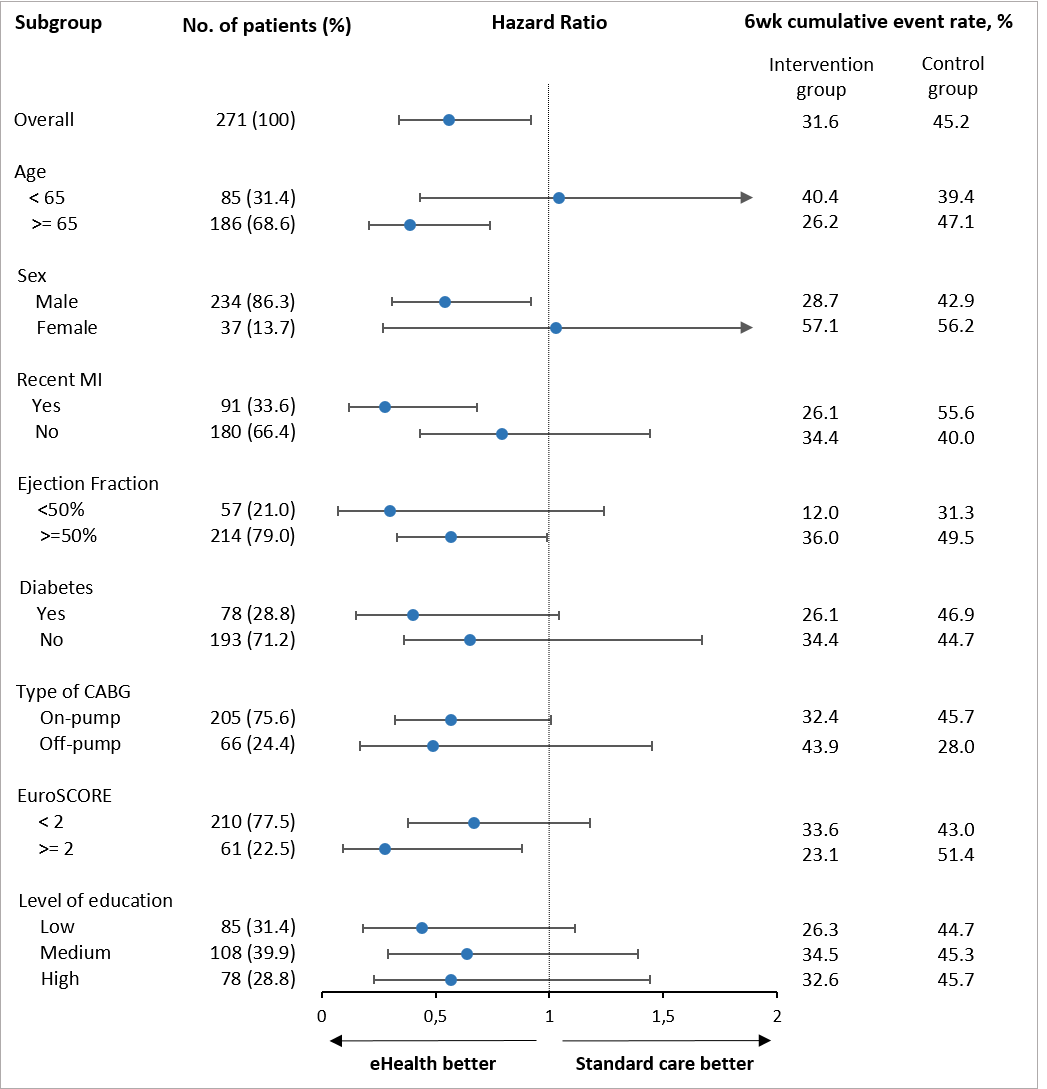
**

## **Figure 1.** Subgroup analysis


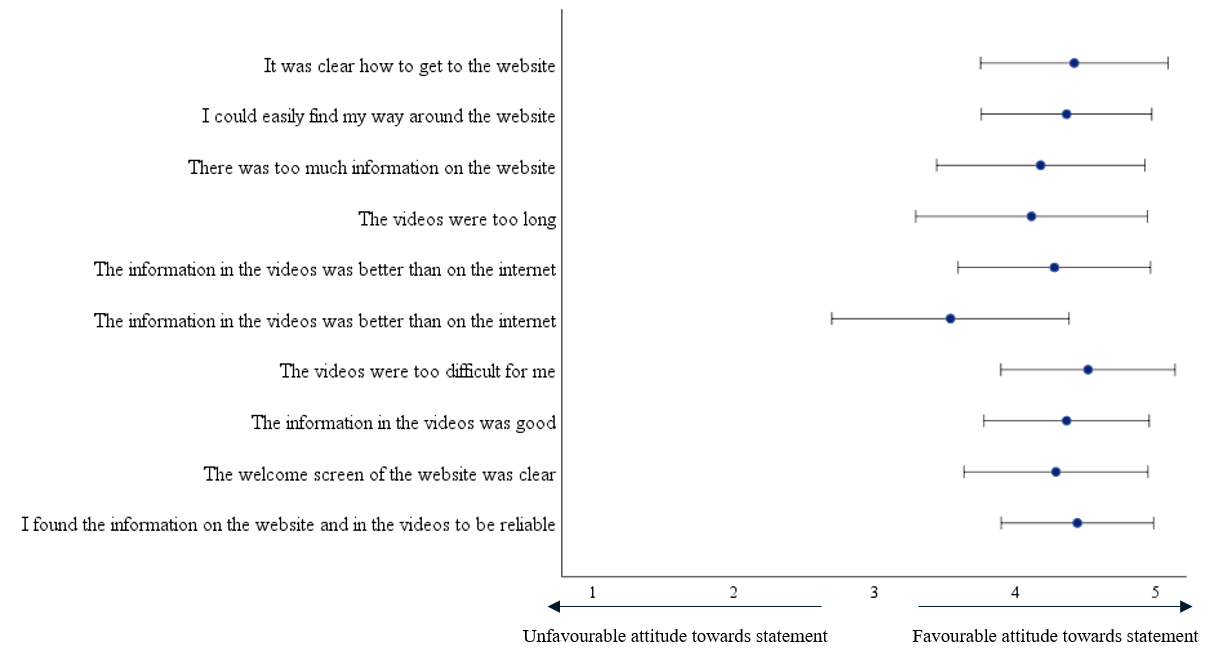


## **Figure 2.** Patient feedback on education videos


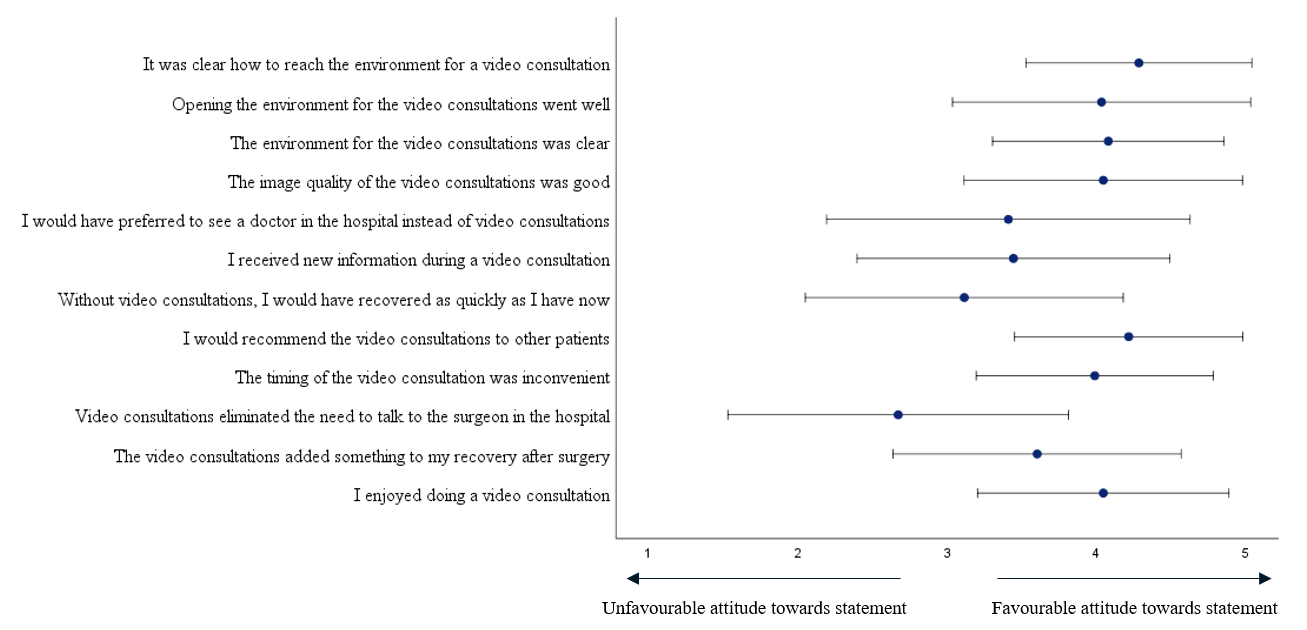


## **Figure 3.** Patient feedback on video consultations
