# Supplementary material for: Effects of Video-Based Patient Education and Consultation on Unplanned Health Care Utilization and Early Recovery After Coronary Artery Bypass Surgery (IMPROV-ED): Randomized Controlled Trial
Source: J Med Internet Res. 2022 Aug 26;24(8):e37728. doi: 10.2196/37728 (PMC9463622; doi:10.2196/37728)
Supplement: Multimedia Appendix 2 [file jmir_v24i8e37728_app2.pdf]

CONSORT-EHEALTH Checklist V1.6.2 Report	Manuscript Number	37728

(based on CONSORT-EHEALTH V1.6), available at [http://tinyurl.com/consort-ehealth-v1-6].

Date completed

8/4/2022 15:44:50

by

Gijs van Steenbergen

Effects of Video-Based Patient Education and Consultation on Unplanned Health Care Utilization and Early Recovery After Coronary Artery Bypass Surgery (IMPROV-ED): Randomized Controlled Trial

TITLE

1a-i) Identify the mode of delivery in the title

Effects of Video-Based Patient Education and Consultation on Unplanned Health Care Utilization and Early Recovery After Coronary Artery Bypass Surgery (IMPROV-ED): Randomized Controlled Trial

1a-ii) Non-web-based components or important co-interventions in title

1a-iii) Primary condition or target group in the title

Effects of Video-Based Patient Education and Consultation on Unplanned Health Care Utilization and Early Recovery After Coronary Artery Bypass Surgery (IMPROV-ED): Randomized Controlled Trial

ABSTRACT

1b-i) Key features/functionalities/components of the intervention and comparator in the METHODS section of the ABSTRACT

Participants in the intervention group had, alongside standard care, access to an eHealth program consisting of online education videos and video consultations developed in conjunction with the Dutch Heart Foundation. The control group received standard care.

1b-ii) Level of human involvement in the METHODS section of the ABSTRACT

1b-iii) Open vs. closed, web-based (self-assessment) vs. face-to-face assessments in the METHODS section of the ABSTRACT

1b-iv) RESULTS section in abstract must contain use data

1b-v) CONCLUSIONS/DISCUSSION in abstract for negative trials

INTRODUCTION

2a-i) Problem and the type of system/solution

After discharge, patients commonly experience anxiety or uncertainty about symptoms or appropriate physical exercise [4]. These issues are typically addressed during hospitalization; however, after discharge, patients' recall of information is often incomplete and they do not always know who to address with questions [4]. The advantages of a shortened hospital stay might therefore be counterbalanced by preventable unplanned health care utilization, especially since planned care is not initiated until several weeks after discharge. At present, nearly 1 in 7 patients are readmitted in the first 30 days after discharge for noncardiac causes and roughly 15% of patients visit the emergency department within 1 month after CABG surgery [5-8]. It was estimated that potentially preventable readmissions following CABG surgery cost Medicare US $151 million in 2005, placing a significant burden on society [7]. With the expected increase in the number of future patients undergoing CABG surgery, this is a pressing issue urging evaluation and a potential redesign of postoperative follow-up.

2a-ii) Scientific background, rationale: What is known about the (type of) system

eHealth is defined by the World Health Organization as "the cost-effective and secure use of information and communication technologies in support of health and health-related fields," which encompasses multiple digital interventions that can aid in the delivery of patient-centered care and postoperative patient guidance, thereby potentially reducing unplanned health care utilization [9]. eHealth strategies have been successfully applied in postoperative follow-up in various forms, which have been shown to improve patient outcomes, speed recovery, and reduce health care utilization in various surgical populations [10]. In addition, eHealth has proven to be of value for patients to enhance their self-management through better understanding of their disease, increased independence, and improved acceptance to adhere to lifestyle advice [3,11]. However, experience with eHealth in patients following CABG surgery is limited, and it remains unclear if eHealth strategies would be effective in this population.

Does your paper address CONSORT subitem 2b?

The objective of this trial was to fill this knowledge and experience gap. We hypothesized that restructuring the postoperative period with an eHealth strategy will reduce unplanned health care utilization through improved mental and physical health and faster recovery.

METHODS

3a) CONSORT: Description of trial design (such as parallel, factorial) including allocation ratio

The IMPROV-ED trial was a randomized controlled trial (RCT)

3b) CONSORT: Important changes to methods after trial commencement (such as eligibility criteria), with reasons

No changes were made to the study protocol between publication and initiation of the trial.

3b-i) Bug fixes, Downtimes, Content Changes

4a) CONSORT: Eligibility criteria for participants

To minimize selection bias, all patients on the waiting list for isolated CABG surgery over 18 years of age were contacted by telephone and informed about the study by one of the investigators. Patients were eligible for participation if they had access to a computer/tablet/smartphone with internet connection and a webcam/built-in camera; had sufficient knowledge of the use of internet and email (assistance was allowed); and were able to speak, read, and interpret the Dutch language. The eHealth strategy would not be applicable to patients who did not comply with these inclusion criteria and they were therefore not eligible for participation.

4a-i) Computer / Internet literacy

4a-ii) Open vs. closed, web-based vs. face-to-face assessments:

Upon randomization, patients in the intervention group received access to the educational videos via a link sent by email. The same link was sent via email again at discharge. By clicking the link, patients were referred to a hidden (for nonparticipants and the control group) part of the website from the Dutch Heart Foundation that contained the educational videos. The content of the educational videos was constructed and validated by physicians and patient representatives prior to the trial. Based on these evaluations and a scoping review of the literature on delivery of information to patients with varying degrees of health literacy, the full content was delivered to patients at inclusion instead of by fragmentized access to videos applicable to the patient's situation [13]. Nevertheless, to prevent cognitive overload in patients with low health literacy, educational videos were categorized in three categories: treatment (10 videos with information on the surgery and how to prepare for admission), recovery (6 videos about what to expect in the postoperative course and when to contact a physician), and healthy living (2 videos on cardiovascular risk management, including smoking cessation, weight reduction, cholesterol management, and exercise). The videos were delivered in spoken text supported by animations for optimal health communication to patients with low and adequate health literacy [13]. Usage data were extracted from the web log for evaluation purposes. Educational videos were available to patients in the intervention group throughout the trial (ie, not only when the link was sent). See the published study protocol for an illustrative overview of the educational videos [11].

4a-iii) Information giving during recruitment

4b) CONSORT: Settings and locations where the data were collected

All patients received questionnaires at inclusion (anxiety subscale of the HADS), at discharge (HADS and RI-10), 1 week after discharge (HADS and RI-10), 2 weeks after discharge (HADS and RI-10), and 6 weeks after discharge (HADS, RI-10, and iMCQ). Only the anxiety subscale from the HADS was used. A higher score indicated more symptoms of anxiety (HADS maximum score 21) or favorable progress of recovery (RI-10 maximum score 50). The iMCQ resulted in absolute frequencies of visits for the questioned care activities. Patients in the intervention group also received a self-made questionnaire to evaluate the eHealth strategy and to question patients about the use of the education videos (see Figures S1 and S2 in Multimedia Appendix 1). If patients had not returned the iMCQ by 8 weeks postdischarge, the questionnaire was conducted over the telephone. If patients had not returned 2 subsequent questionnaires, a research nurse called patients with a reminder. Questionnaires that were not returned or collected otherwise were considered missing.

4b-i) Report if outcomes were (self-)assessed through online questionnaires

see awnser to previous question

4b-ii) Report how institutional affiliations are displayed

5) CONSORT: Describe the interventions for each group with sufficient details to allow replication, including how and when they were actually administered

5-i) Mention names, credential, affiliations of the developers, sponsors, and owners

5-ii) Describe the history/development process

**5-iii) Revisions and updating**		
**5-iv) Quality assurance methods**		
**5-v) Ensure replicability by publishing the source code, and/or providing screenshots/screen-capture video, and/or providing flowcharts of the algorithms used**		
**5-vi) Digital preservation**		
**5-vii) Access**		
Upon randomization, patients in the intervention group received access to the educational videos via a link sent by email. The same link was sent via email again at discharge. By clicking the link, patients were referred to a hidden (for nonparticipants and the control group) part of the website from the Dutch Heart Foundation that contained the educational videos.		
**5-viii) Mode of delivery, features/functionalities/components of the intervention and comparator, and the theoretical framework**		
The content of the educational videos was constructed and validated by physicians and patient representatives prior to the trial. Based on these evaluations and a scoping review of the literature on delivery of information to patients with varying degrees of health literacy, the full content was delivered to patients at inclusion instead of by fragmentized access to videos applicable to the patient's situation [13]. Nevertheless, to prevent cognitive overload in patients with low health literacy, educational videos were categorized in three categories: treatment (10 videos with information on the surgery and how to prepare for admission), recovery (6 videos about what to expect in the postoperative course and when to contact a physician), and healthy living (2 videos on cardiovascular risk management, including smoking cessation, weight reduction, cholesterol management, and exercise). The videos were delivered in spoken text supported by animations for optimal health communication to patients with low and adequate health literacy		
**5-ix) Describe use parameters**		
**5-x) Clarify the level of human involvement**		
**5-xi) Report any prompts/reminders used**		
If patients had not returned 2 subsequent questionnaires, a research nurse called patients with a reminder. Questionnaires that were not returned or collected otherwise were considered missing.		
**5-xii) Describe any co-interventions (incl. training/support)**		
Upon randomization, patients in the intervention group received access to the educational videos via a link sent by email. The same link was sent via email again at discharge. By clicking the link, patients were referred to a hidden (for nonparticipants and the control group) part of the website from the Dutch Heart Foundation that contained the educational videos. The content of the educational videos was constructed and validated by physicians and patient representatives prior to the trial. Based on these evaluations and a scoping review of the literature on delivery of information to patients with varying degrees of health literacy, the full content was delivered to patients at inclusion instead of by fragmentized access to videos applicable to the patient's situation [13]. Nevertheless, to prevent cognitive overload in patients with low health literacy, educational videos were categorized in three categories: treatment (10 videos with information on the surgery and how to prepare for admission), recovery (6 videos about what to expect in the postoperative course and when to contact a physician), and healthy living (2 videos on cardiovascular risk management, including smoking cessation, weight reduction, cholesterol management, and exercise). The videos were delivered in spoken text supported by animations for optimal health communication to patients with low and adequate health literacy [13]. Usage data were extracted from the web log for evaluation purposes. Educational videos were available to patients in the intervention group throughout the trial (ie, not only when the link was sent). See the published study protocol for an illustrative overview of the educational videos [11].		
**6a) CONSORT: Completely defined pre-specified primary and secondary outcome measures, including how and when they were assessed**		
The primary outcomes of the IMPROV-ED trial were the volume and costs of unplanned health care utilization as defined as a composite of all emergency department visits, outpatient clinic visits, rehospitalization, patient-initiated telephone consultations, and visits to a general practitioner as measured by the Institute for Medical Technology Assessment Medical Consumption Questionnaire (iMCQ) at the 6-week follow-up [14]. Cross-validation with the patients' reported health care utilization was performed by contacting their health care providers. The secondary outcomes were the individual unplanned health care activities and a composite of planned and unplanned in-hospital care (emergency department visits, outpatient clinic visits, rehospitalization, and patient-initiated telephone consultations) and planned and unplanned primary care (consultations with a general practitioner, allied health professionals, psychologists) at 6 weeks. The other secondary outcomes were the patients' self-reported physical and mental health, as measured with the Hospital Anxiety and Depression Scale (HADS) and Recovery Index-10 (RI-10) questionnaires [15,16].		
**6a-i) Online questionnaires: describe if they were validated for online use and apply CHERRIES items to describe how the questionnaires were designed/deployed**		
**6a-ii) Describe whether and how "use" (including intensity of use/dosage) was defined/measured/monitored**		
**6a-iii) Describe whether, how, and when qualitative feedback from participants was obtained**		
**6b) CONSORT: Any changes to trial outcomes after the trial commenced, with reasons**		
All patients received questionnaires at inclusion (anxiety subscale of the HADS), at discharge (HADS and RI-10), 1 week after discharge (HADS and RI-10), 2 weeks after discharge (HADS and RI-10), and 6 weeks after discharge (HADS, RI-10, and iMCQ). Only the anxiety subscale from the HADS was used. A higher score indicated more symptoms of anxiety (HADS maximum score 21) or favorable progress of recovery (RI-10 maximum score 50). The iMCQ resulted in absolute frequencies of visits for the questioned care activities. Patients in the intervention group also received a self-made questionnaire to evaluate the eHealth strategy and to question patients about the use of the education videos (see Figures S1 and S2 in Multimedia Appendix 1). If patients had not returned the iMCQ by 8 weeks postdischarge, the questionnaire was conducted over the telephone. If patients had not returned 2 subsequent questionnaires, a research nurse called patients with a reminder. Questionnaires that were not returned or collected otherwise were considered missing.		
**7a) CONSORT: How sample size was determined**		
**7a-i) Describe whether and how expected attrition was taken into account when calculating the sample size**		
**7b) CONSORT: When applicable, explanation of any interim analyses and stopping guidelines**		
The primary outcomes of the IMPROV-ED trial were the volume and costs of unplanned health care utilization as defined as a composite of all emergency department visits, outpatient clinic visits, rehospitalization, patient-initiated telephone consultations, and visits to a general practitioner as measured by the Institute for Medical Technology Assessment Medical Consumption Questionnaire (iMCQ) at the 6-week follow-up [14]. Cross-validation with the patients' reported health care utilization was performed by contacting their health care providers. The secondary outcomes were the individual unplanned health care activities and a composite of planned and unplanned in-hospital care (emergency department visits, outpatient clinic visits, rehospitalization, and patient-initiated telephone consultations) and planned and unplanned primary care (consultations with a general practitioner, allied health professionals, psychologists) at 6 weeks. The other secondary outcomes were the patients' self-reported physical and mental health, as measured with the Hospital Anxiety and Depression Scale (HADS) and Recovery Index-10 (RI-10) questionnaires [15,16].		
**8a) CONSORT: Method used to generate the random allocation sequence**		
To minimize selection bias, all patients on the waiting list for isolated CABG surgery over 18 years of age were contacted by telephone and informed about the study by one of the investigators.		
**8b) CONSORT: Type of randomisation; details of any restriction (such as blocking and block size)**		
At inclusion, patients were randomized 1:1 to the intervention or control group using a block size of 4.		
**9) CONSORT: Mechanism used to implement the random allocation sequence (such as sequentially numbered containers), describing any steps taken to conceal the sequence until interventions were assigned**		
A certified program was used for sequence generation and randomization (Research Manager).		
**10) CONSORT: Who generated the random allocation sequence, who enrolled participants, and who assigned participants to interventions**		
To minimize selection bias, all patients on the waiting list for isolated CABG surgery over 18 years of age were contacted by telephone and informed about the study by one of the investigators.		
**11a) CONSORT: Blinding - If done, who was blinded after assignment to interventions (for example, participants, care providers, those assessing outcomes) and how**		
**11a-i) Specify who was blinded, and who wasn't**		
The nurse practitioner/junior doctor who conducted the VCs was blinded to the study's objectives and outcomes. Study participants were not blinded.		
**11a-ii) Discuss e.g., whether participants knew which intervention was the "intervention of interest" and which one was the "comparator"**		
**11b) CONSORT: If relevant, description of the similarity of interventions**		
not applicable/relevant for study		
**12a) CONSORT: Statistical methods used to compare groups for primary and secondary outcomes**		

The main analysis was performed according to the intention-to-treat (ITT) principle. Because patients in the intervention group were not obliged to use the educational videos and VCs might not be possible due to technical errors, per-protocol analysis was also performed, which included only patients who used the intervention strategy as intended (defined as having at least one VC or TC and accessed the educational videos at least once).
Planned subgroup analyses of the primary outcome were performed according to age (<65 years vs ≥65 years), sex, recent myocardial infarction, left ventricular function, diabetes, type of CABG (on-pump vs off-pump), log EuroScore, and highest level of education.
Continuous variables and outcomes are expressed as mean (SD) in cases of a normal distribution and as median (IQR) in cases of a nonnormal distribution. The Kolmogorov-Smirnov test and Q-Q plots were used to test for normality of the data distribution. Categorical data are summarized as absolute and relative frequencies. The updated Dutch Manual for Cost Analysis in Health Care Research was used as the source for cost prices per health care activity if available [19]. Other tariffs were calculated using top-down microcosting as described by Tan et al [20] (see Multimedia Appendix 1 for details). Each consumed health care activity was multiplied by the cost price and total costs were calculated by summing these multiplications. The HADS and RI-10 questionnaire scores at each interval were compared between study groups. P<.05 was considered significant. Primary and secondary outcomes are presented with effect-size estimates and 95% CIs using the Cox proportional hazards model. The proportional hazard assumption was assessed by log (–log) plots. Analyses were performed using SPSS 25 and RStudio.

12a-i) Imputation techniques to deal with attrition / missing values

not applicable/relevant for study

12b) CONSORT: Methods for additional analyses, such as subgroup analyses and adjusted analyses

see awnser to previous questions

RESULTS

13a) CONSORT: For each group, the numbers of participants who were randomly assigned, received intended treatment, and were analysed for the primary outcome

In total, 280 patients were included in the study between February 2020 and December 2021, and subsequently randomized yielding 140 patients in each study group. One patient in the intervention group and two patients in the control group were excluded after randomization because they underwent percutaneous coronary intervention instead of CABG surgery. In the intervention group, three patients were lost to follow-up (1 withdrew consent, 1 had an early readmission due to a complication, and 1 died). In the control group, three patients were lost to follow-up (1 withdrew consent and 2 died). The ITT analysis therefore consisted of 136 and 135 patients in the intervention and control group, respectively. Weblog and planning data revealed that 8 patients did not use the intervention as intended, whereby 128 patients were included in the intervention group in the per-protocol analysis (Figure 1).

13b) CONSORT: For each group, losses and exclusions after randomisation, together with reasons

see awnsers to previous questions

13b-i) Attrition diagram

14a) CONSORT: Dates defining the periods of recruitment and follow-up

see awnsers to previous questions

14a-i) Indicate if critical “secular events” fell into the study period

14b) CONSORT: Why the trial ended or was stopped (early)

see awnsers to previous questions

15) CONSORT: A table showing baseline demographic and clinical characteristics for each group

Baseline characteristics of patients were similar in the two groups (Table 1), with a median age of 67.9 and 69.6 years for the intervention and control group, respectively. The majority of patients were male in both groups. At the time of surgery, 25% of patients had an urgent indication and the remainder underwent surgery in the elective setting. In the majority of patients, on-pump CABG was performed using 3 distal anastomoses. The left or right internal mammary artery was used in >98% of patients. Duration of admission was also similar in the two groups (Table 1).

15-i) Report demographics associated with digital divide issues

not applicable/relevant for study

16a) CONSORT: For each group, number of participants (denominator) included in each analysis and whether the analysis was by original assigned groups

16-i) Report multiple “denominators” and provide definitions

See awnsers to previous questions

16-ii) Primary analysis should be intent-to-treat

17a) CONSORT: For each primary and secondary outcome, results for each group, and the estimated effect size and its precision (such as 95% confidence interval)

OutcomeseHealth group
(n=136)
Standard care (n=135)
Hazard ratio (95% CI)
P value

Primary outcomes

Composite outcomea, n (%)43 (31.6)61 (45.2)0.56 (0.34 – 0.92).022

Cost (Eurob), Median (IQR)0 (0-95)
66 (0-215)
N/Ac<.001

Cost (Euro), mean (SD)183 (515)
285 (777)
N/A<.001

Secondary outcomes, n (%)

Composite unplanned in-hospital care36 (26.5)53 (39.3)0.56 (0.33-0.93)
.026

Emergency department visits14 (10.3)23 (17.0)0.56 (0.27-1.14)
.11

Readmissions7 (5.1)9 (6.7)0.76 (0.28-2.10)
.59

Outpatient clinic visits11 (8.1)10 (7.4)1.10 (0.45-2.68)
.83

Telephonic consultations29 (21.3)47 (34.8)0.51 (0.29-0.87)
.014

General practitioner visits (unplanned)28 (20.6)41 (30.4)0.59 (0.34-1.04)
.066

Composite of all in-hospital cared69 (50.7)97 (71.9)0.40 (0.24-0.67)
<.001

Composite of all primary caree82 (60.3)101 (74.8)0.58 (0.36-0.97)
.037

17a-i) Presentation of process outcomes such as metrics of use and intensity of use

**17b) CONSORT: For binary outcomes, presentation of both absolute and relative effect sizes is recommended**		
see awnsers to previous questions		
**18) CONSORT: Results of any other analyses performed, including subgroup analyses and adjusted analyses, distinguishing pre-specified from exploratory**		
see supplementary file		
**18-i) Subgroup analysis of comparing only users**		
**19) CONSORT: All important harms or unintended effects in each group**		
not applicable/relevant for study		
**19-i) Include privacy breaches, technical problems**		
**19-ii) Include qualitative feedback from participants or observations from staff/researchers**		
DISCUSSION		
**20) CONSORT: Trial limitations, addressing sources of potential bias, imprecision, multiplicity of analyses**		
**20-i) Typical limitations in ehealth trials**		
However, health care utilization is the resultant of a multifactorial behavioral model that attributes a combination of predisposing factors (eg, patient characteristics such as age, sex, sociodemographic parameters, or health literacy and attitude toward health), enabling factors (eg, income, health insurance status, health care organization), and need factors (eg, experience with health care) to health care utilization [28]. The eHealth strategy used in the IMPROV-ED trial has a positive influence on some of these attributes but not all. Interestingly, subgroup analysis showed that the eHealth program had a greater benefit in more vulnerable patients (EuroScore≥2) and revealed a trend toward more benefit in patients with a low level of education. By contrast, a small group of patients who provided informed consent did not use the educational videos or VCs that were part of the eHealth strategy. These patients reported to have received sufficient information from their physician, nurse, or paramedic during admission, or that they found the relevant information online themselves. It might therefore be reasonable to consider adding different modes of digital health delivery to the currently used eHealth strategy (eg, mobile apps, live chat, home monitoring, telerehabilitation) to manage more attributes of health utilization and to offer a more individualized approach tailored to the patients' needs. Combining different modes of digital care might thereby further reduce health care utilization and potentially also improve clinical outcomes [21].		
**21) CONSORT: Generalisability (external validity, applicability) of the trial findings**		
**21-i) Generalizability to other populations**		
**21-ii) Discuss if there were elements in the RCT that would be different in a routine application setting**		
**22) CONSORT: Interpretation consistent with results, balancing benefits and harms, and considering other relevant evidence**		
**22-i) Restate study questions and summarize the answers suggested by the data, starting with primary outcomes and process outcomes (use)**		
The principal finding of the IMPROV-ED trial is that an eHealth strategy comprising educational videos and VCs results in a reduction of unplanned care and costs. In addition, the eHealth strategy is associated with faster patient-reported recovery. These findings are of medical and societal importance given the increasing interest in digital health and the need for value-based alongside evidence-based care. Our study is the first to provide robust evidence that an eHealth intervention can aid in reduction of health care utilization and associated costs. This effect appears applicable to both in-hospital care as well as primary care. One of the most pressing concerns from health care insurance companies and decision-makers toward eHealth is the great investment that is required for development of content and the necessary infrastructure and issues that arise after implementation due to lack of reimbursement options [21]. Our findings refute these concerns by showing positive effects on costs. Furthermore, the eHealth strategy did not only contribute to less patients consuming care (Table 2) but also reduced the care consumed per patient (Table S1 of Multimedia Appendix 1), which underlines the high potential of eHealth strategies for this patient population to also positively influence the burden on health care personnel. With an aging population, a vast increase in health care consumption is expected in the near future. Based on the results of our study, an eHealth program is proven to aid in the sustainment of health care systems.		
**22-ii) Highlight unanswered new questions, suggest future research**		
Other information		
**23) CONSORT: Registration number and name of trial registry**		
Trial Registration: Netherlands Trial Registry NL8510; www.trialregister.nl		
**24) CONSORT: Where the full trial protocol can be accessed, if available**		
A detailed study protocol was published prior to enrollment of the first study participant [11].		
**25) CONSORT: Sources of funding and other support (such as supply of drugs), role of funders**		
Funding: not applicable		
**X26-i) Comment on ethics committee approval**		
**x26-ii) Outline informed consent procedures**		
**X26-iii) Safety and security procedures**		
**X27-i) State the relation of the study team towards the system being evaluated**		
